# New exploration of KRAS^G12D^ inhibitors and the mechanisms of resistance

**DOI:** 10.1186/s40164-025-00637-4

**Published:** 2025-03-17

**Authors:** Ying Li, Junfeng Zhao, Yintao Li

**Affiliations:** 1 Department of Respiratory Oncology, Shandong Cancer Hospital and Institute, Shandong First Medical University, Shandong Academy of Medical Sciences, 440 Jiyan Road, Huaiyin District, Jinan, Shandong 250000 China; 2 Department of Radiation Oncology, Shandong Cancer Hospital and Institute, Shandong First Medical University, Shandong Academy of Medical Sciences, Jinan, Shandong 250000 China

**Keywords:** HRS-4642, KRAS G12D, KRAS G12R, KRAS Inhibition, Resistance

## Abstract

The development of Kirsten rat sarcoma viral oncogene homologue (KRAS) targeted therapies has been the focus of cancer treatment. The most common mutant subtypes of KRAS driver genes are *G12C*, *G12V*, and *G12D*, and are associated with poor prognosis. Up to now, inhibitors specifically targeting KRAS^G12D^ mutant proteins are all in the pre-clinical/early clinical research stage, and there is still a lack of effective clinical targeting strategies. In their recently published article, Zhou et al. developed a high-affinity, selective, long-acting, non-covalent KRAS^G12D^-specific inhibitor and, further combined with the proteasome inhibitor carfilzomib, found that this protocol can achieve the purpose of killing mutant cell lines and inhibiting tumor growth in vitro and in vivo. Here, we aim to describe a potential novel therapy for patients with KRAS^G12D^ mutations and present the first KRAS^G12D^-specific inhibitor to be proven as clinically effective. Different mutations of KRAS gene and mechanisms of KRAS drug resistance were also discussed.

Kirsten rat sarcoma viral oncogene homologue (KRAS) is the most frequently mutated oncogene in human cancer, among which the most common mutations are subtypes *G12C, G12V, G12D* and are associated with poor prognosis [1, 2]. KRAS has long been considered difficult to be used as a drug target, but recent advances in related fields have made the development of KRAS targeted drugs a reality. Selective inhibitors targeting the KRAS^G12C^ mutation have been successively approved. More KRAS targeted therapeutic strategies are still under development.

## KRASG12D inhibitor - HRS-4642

KRAS^G12D^ is commonly detected in pancreatic ductal adenocarcinoma, colorectal cancer, and lung adenocarcinoma [3]. Since the KRAS^G12D^ mutant protein lacks an active residue close to the switch-II binding pocket, covalent inhibitors of the KRAS^G12C^ mutation can no longer be applied in drug design [3]. It is more challenging to develop KRAS^G12D^ inhibitors than to develop KRAS^G12C^ inhibitors. The important inhibitors specifically targeting KRAS^G12D^ mutant protein (Table 1), such as MRTX1133, GFH375 and AST2169, are in the preclinical clinical research stage, and there is still a lack of effective targeting strategies in clinical practice.

**Fig. 1:**
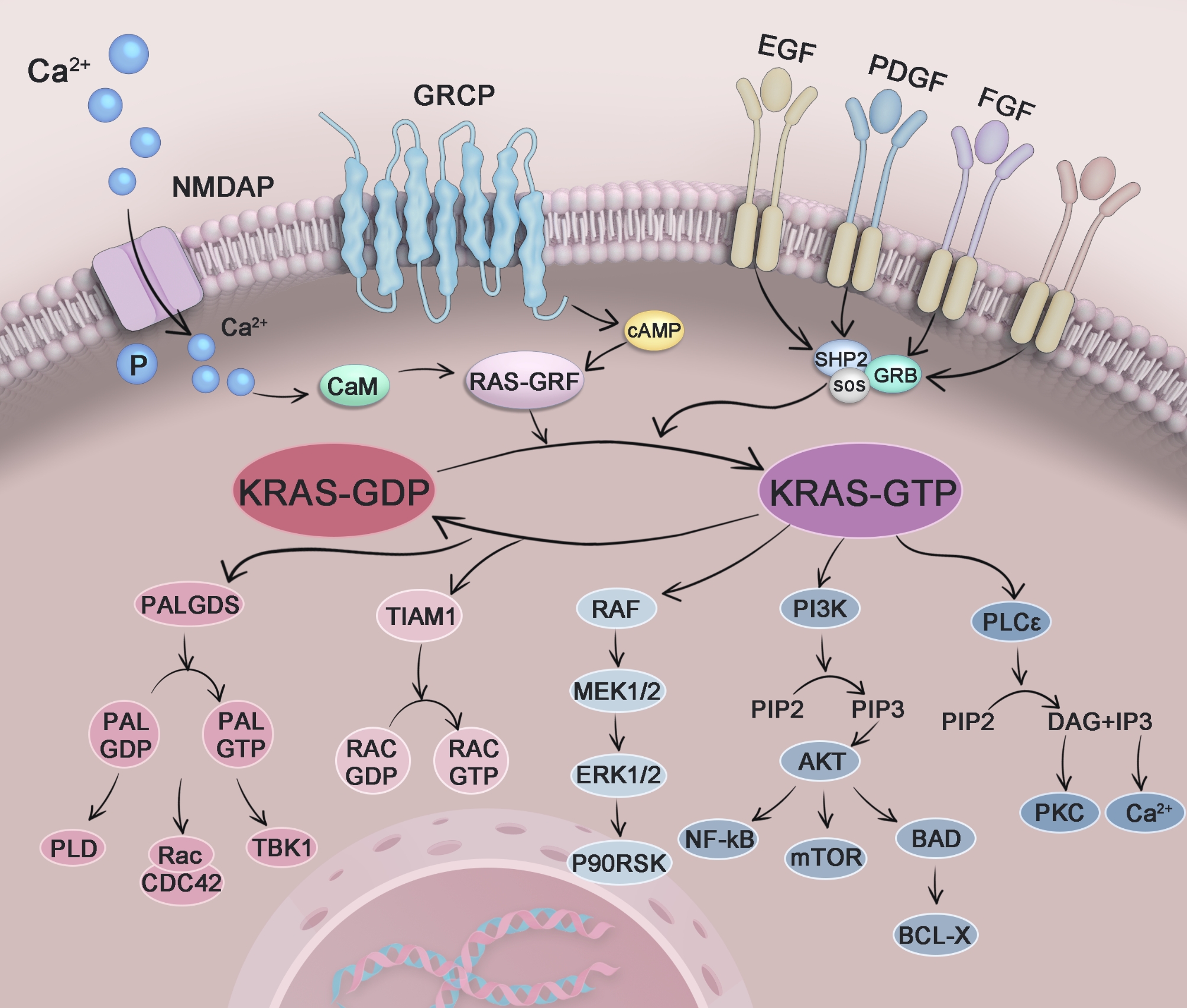
HRS-4642 can inhibit KRAS G12D binding to SOS or RAF1 and inhibit downstream MEK-ERK signaling pathways KRAS: Kirsten rat sarcoma viral oncogene homologue SOS: Guanine nucleotide exchange factors (GEFs, e.g., SOS proteins) RAF: Mitogen-Activated Protein Kinase Kinase MEK: Mitogen-activated protein kinase kinase ERK: Extracellular signaling-related kinases KRAS inhibitor resistance is strongly associated with activation of the PI3K-AKT-mTOR signaling pathway

Zhou et al. presented the first clinically effective KRAS^G12D^-specific inhibitor, HRS-4642, and a novel therapeutic strategy involving combination with proteasome inhibitors [4]. HRS-4642 is a high-affinity, selective, long-acting, non-covalent KRAS^G12D^ inhibitor that has demonstrated strong efficacy against KRAS^G12D^ mutant cancers both in vivo and in vitro. In vitro surface plasmon resonance experiments have shown that the equilibrium dissociation constant of HRS-4642 and KRAS^G12D^ was 21 and 17 times lower than those of KRAS^G12C^ and wild-type KRAS protein, respectively. This indicates that HRS-4642 has high selectivity and potency against KRAS^G12D^. Binding inhibition experiments showed that HRS-4642 inhibited the binding of KRAS^G12D^ to SOS1 or RAF1 (Fig. 1), which played a dual-blocking role and inhibited the downstream MEK-ERK signaling pathway. Additionally, using 16 human cells with different KRAS mutation states, HRS-4642 showed strong specific inhibition of KRAS^G12D^ mutant cell lines. In vivo experiments using human pancreatic cancer AsPC-1 cells, human colorectal cancer GP2d xenograft tumor models, and lung adenocarcinoma PDX models with the KRAS^G12D^ mutation have confirmed that HRS-4642 can significantly inhibit KRAS^G12D^ tumor growth, has good pharmacokinetic and pharmacodynamic characteristics, and tends to accumulate in tumors. This shows that HRS-4642 is expected to have a strong inhibitory effect on cell proliferation and tumor growth. Given the encouraging results of the preclinical studies, investigators initiated the first Phase I clinical study in humans. Nine patients with non-small cell lung cancer were included. After treatment with HRS-4642, two patients experienced a partial response while seven patients had stable disease. Specifically, two patients achieved a partial response after treatment with 200 and 300 mg HRS-4642 once a week. The representative lesion in Patient 1 disappeared after 6 weeks of treatment, the pleural effusion subsided, and the target lesion shrank by 53%. The number of major lesions in Patient 2 reduced by 31%. These findings indicated that HRS-4642 has good antitumor activity and offers an effective treatment strategy for patients with KRAS^G12D^ tumor, marking a new stage of targeted therapy for KRAS^G12D^.

KRAS plays a critical role as the central regulator of signal transduction. Most patients do not show a treatment response to KRAS inhibitors in clinical practice; instead, they only show moderate anti-tumor efficacy, and disease progression is inevitable. To enhance the treatment efficacy and delay the emergence of resistance to targeted therapies, genome-wide CRISPR-Cas9 knockout screening was used to screen ≈ 19,000 genes and map the sensitization and resistance spectrum of HRS-4642. The discovery of potential targets for sensitization to HRS-4642, including Aurora A and Pi3k, was consistent with previous reports on KRAS^G12C^ inhibitors [5, 6]. Enrichment analysis of the screening data revealed that the proteasome might be a sensitization target for HRS-4642, which is consistent with the synergistic effect observed in the KRAS^G12C^ mutant cell line, indicating that the proteasome pathway is crucially involved in KRAS mutations [7]. Analysis of The Cancer Genome Atlas data also revealed that low proteasome signature expression is associated with prolonged survival in pancarcinoma, lung adenocarcinoma, and pancreatic cancer. The proteasome inhibitors carfilzomib and HRS-4642 were used to conduct in vitro and in vivo experiments, and it was found that the regimen could synergistically kill KRAS^G12D^ mutant cell lines in vitro and inhibit KRAS^G12D^ tumor growth in vivo. RNA-seq and DIA protein profiling revealed that combination therapy using HRS-4642 and carfilzomib exerts synergistic anti-tumor effects mainly by down- and up-regulating the Notch4 and IFNα signaling pathways, respectively.

Additionally, KRAS not only induces tumor cell proliferation but also affects the immune microenvironment, thereby promoting tumor progression [8–10]. Flow cytometry and immune profiling assays revealed that HRS-4642 alone or in combination with carfilzomib significantly promoted the invasion and activation of CD4 + and CD8 + T cells in KRAS^G12D^ tumors as well as anti-tumor immune cells in the tumor immune microenvironment. It provides a theoretical basis for “target-immune combination” for KRAS^G12D^ tumors.

This study describes a potential novel therapy for patients with KRAS^G12D^ mutations and presents the first KRAS^G12D^-specific inhibitor to be proven as clinically effective. This provides strong preclinical evidence for the combination of HRS-4642 with proteasome inhibitors or immune checkpoint inhibitors. The possibility of clinical application of these combination strategies will be further explored in the future, which may provide an effective and safe targeted combination strategy for patients with KRAS^G12D^ mutant solid tumors.

**Table 1:** KRAS^G12D^ inhibitors are under development

KRAS^G12D^ Inhibitor	Mechanism of Drug action		Clinical stage
HRS-4642	KRAS^G12D^ Inhibitor	HRS-4642 inhibits the binding of KRAS^G12D^ to SOS1 or RAF1, thereby inhibiting the downstream MEK-ERK signaling pathway	Phase I/II clinical study
MRTX1133	Non-covalently bonded KRAS^G12D^ selective inhibitor	MRTX1133 inhibits KRAS^G12D^ inactive and active states	Phase I clinical study
GFH375(VS-7375)	KRAS^G12D^ (ON/OFF) Inhibitor	GFH375 has a unique ON/OFF binding mechanism and can act on both activated (GTP-binding) and inactivated (GDP-binding) states of KRAS^G12D^ mutant protein. GFH375 binds to KRAS G12D protein in a non-covalent form, thereby inhibiting its binding to downstream effector proteins and suppressing their pathway activation, and ultimately inhibiting tumor cell proliferation to achieve anti-tumor effects.	Phase I/II clinical study
AST2169	KRAS^G12D^ Inhibitor (Liposome)	AST2169 liposome inhibits the function of KRAS G12D mutant protein, down-regulates the activity of related signaling pathways, effectively prevents cell cycle progression, induces apoptosis, and thus reduces the proliferation rate of tumor cells to achieve anti-tumor effects.	Phase I clinical study
RMC-9805	Covalent mutation-selective KRAS inhibitors	RMC-9805 selectively covalently modifies Asp-12 and interferes with RAS downstream signaling by forming a ternary complex with cyclophilin A (CypA) and the “ON” state of RASG12.	Phase I/II clinical study
ASP3082	KRAS^G12D^ protein degrader	ASP3082 is a novel small-molecule targeted protein degradation chimera that binds to and selectively targets KRAS^G12D^ mutant protein for degradation by recruiting an E3 ubiquitin ligase protein.	Phase I clinical study

## Pancreatic ductal adenocarcinoma specific mutant KRASG12R

Pancreatic ductal adenocarcinoma (PDAC) is one of the deadliest cancers [11]. Most patients with PDAC are diagnosed at an advanced stage or after metastasis, and it can be removed surgically in only 20% of the cases [11]. KRAS gene mutations are very common in PDAC, and different KRAS mutations may vary in the degree of tumor malignancy. At present, the research on KRAS^G12R^ and KRAS^G12V^ is not comprehensive, and its effect on patient survival has not been fully evaluated. McIntyre et al. comprehensively analyzed KRAS-associated mutations in PDAC patients and found that KRAS^G12R^ mutations were significantly enriched in early (stage I) PDAC [11]. Compared with KRAS^G12D^, KRAS^G12R^ mutant PDAC had fewer distant relapses and improved overall survival. This study reveals the key role of mutation-specific analysis in prognostic assessment and provides important clues to understanding the biological mechanisms of PDAC.

### Mechanism of KRAS inhibitor resistance

Although KRAS inhibitors can show some clinical efficacy, the problem of resistance is widespread and the mechanism is not fully understood. Therefore, there is an urgent need to understand the mechanisms of response and resistance to KRAS inhibitors to guide the effective use of these therapies and improve clinical outcomes for patients.

Dilly et al. investigated the mechanism of resistance to KRAS targeted inhibitors in pancreatic cancer. The mechanism of resistance to KRAS inhibitors was comprehensively shared through clinical samples (ctDNA), and multiple in vitro and in vivo models, such as cell lines, organoids, and genetically engineered mouse models included multimodal data at the genome, transcriptome, and protein levels [12]. It was found that in patient samples, drug resistance was closely related to *PIK3CA* and *KRAS* mutations, as well as amplification of *KRASG12C*,* MYC*,* EGFR*, and other genes. In cell lines and organoid models, drug resistance is closely associated with activation of the epithelial-to-mesenchymal transition (EMT) and PI3K-AKT-mTOR signaling pathways. In KPC (Kras^LSL−G12D/+^; Trp53^LSL − R172H/+^; p48-Cre) mouse model, MRTX1133 showed significant tumor inhibition at the beginning of treatment, but resistance eventually emerged. Drug resistance was accompanied by amplification of *Kras*,* Yap1*,* Myc*, and *Cdk6* genes. Drug-resistant tumors exhibit a partial-EMT (pEMT) state, or partial epithelial-mesenchymal transformation, which is a hybrid state along the epithelial-mesenchymal axis. In mouse models, KRAS^G12D^ inhibitors combined with chemotherapy (such as gemcitabine/albumin-bound paclitaxel) significantly delayed tumor recurrence. Removal of EGF and FGF from organoid media can improve the sensitivity to KRAS inhibitors, suggesting that receptor tyrosine kinase (RTK) signaling is a key driver of resistance.

This study combined clinical samples, cell models, organoids, and genetically engineered mouse models to demonstrate the broad applicability of the findings. Single-cell RNA sequencing and ctDNA techniques were used to reveal the dynamic changes of the genome and transcriptome during drug resistance. These findings are based directly on patient samples from clinical trials and provide valuable insights into clinical practice. It systematically integrates genetic and non-genetic mechanisms to reveal the complexity of KRAS inhibitor resistance and provides a theoretical basis for developing combined treatment strategies in the future.

## Conclusion

Targeting KRAS has been considered quite challenging. However, recent advances in medicinal chemistry have allowed KRAS inhibitors to be developed and have shown promising therapeutic efficacy in clinical trials. In the future, further exploration is required to deeply clarify the mechanism of action and drug resistance of different KRAS driver gene mutation subtypes and find more effective and accurate treatment plans to achieve the purpose of individualized treatment.

## Data Availability

No datasets were generated or analysed during the current study.

## Abbreviations

KRAS: Kirsten rat sarcoma viral oncogene homologue
PDAC: pancreatic ductal adenocarcinoma
EMT: epithelial-to-mesenchymal transition
pEMT: partial-EMT
RTK: receptor tyrosine kinase
